# Association between sensory impairment and sarcopenia in older Chinese adults: a 4-Year longitudinal study

**DOI:** 10.1186/s12877-024-05642-6

**Published:** 2025-02-11

**Authors:** Chunjie Huang, Xiaoqing He

**Affiliations:** School of Public Administration, Sichuan University, Chengdu, China

**Keywords:** CHARLS, Hearing impairments, Sarcopenia, Sensory impairment, Visual impairments

## Abstract

**Objectives:**

Sarcopenia is a common geriatric syndrome that significantly increases the risk of falls, fractures, disability, and death in older adults. Sensory impairments are also prevalent among the elderly and may exacerbate the decline in physical function, even affecting muscle health. Understanding whether sensory impairments are risk factors affecting sarcopenia in older adults is crucial for developing effective public health policies and intervention strategies. Therefore, this study aims to explore the association between sensory impairments and sarcopenia and its components.

**Methods:**

This study, based on the Chinese Health and Retirement Longitudinal Study (CHARLS), included 4,195 participants aged 60 and above. The assessment of sensory impairment was based on self-reported visual and hearing capabilities. The diagnosis of sarcopenia followed the consensus of the Asian Working Group on Sarcopenia (AWGS) from 2019. Data analysis was conducted using an ordered logistic regression model, and the results report the odds ratios (ORs) and their 95% confidence intervals (CI).

**Results:**

Single sensory impairments at baseline showed no significant correlation with sarcopenia four years later, while dual sensory impairments (DSI) at baseline were significantly associated with sarcopenia (ORs: 1.308, 95% CI: 1.126–1.519). In the analysis of trends over time, transitions from no sensory impairments (NSI) to DSI (ORs: 1.372, 95% CI: 1.028–1.830), from hearing impairments (HI) to DSI (ORs: 1.334, 95% CI: 1.002–1.778), and persistent DSI (ORs: 1.470, 95% CI: 1.159–1.864) were all significantly associated with sarcopenia. Additionally, we found DSI is associated with poor physical performance and muscle mass but not muscle strength.

**Conclusions:**

Our study indicates that DSI have a more severe impact on sarcopenia compared to single sensory impairments. Our findings offer a new perspective for prevention and intervention strategies, suggesting the inclusion of sensory impairment assessments in the clinical evaluation of sarcopenia risk. For elderly individuals with DSI, comprehensive intervention measures should be provided, such as sensory rehabilitation, nutritional support, and guidance on physical activities. For those with only a single sensory impairment, proactive preventive measures should be taken to prevent the progression to DSI.

**Supplementary Information:**

The online version contains supplementary material available at 10.1186/s12877-024-05642-6.

## Introduction

Sarcopenia is a systemic skeletal muscle disorder that develops with aging, characterized by the progressive decline of muscle strength, mass, and function [1, 2]. In October 2016, sarcopenia was officially classified as an independent disease in the International Classification of Diseases (ICD-10-CM) [3]. In 2016, the number of sarcopenia cases in Europe was estimated at 10.87 million, projected to increase to 18.74 million by 2045, representing a growth rate of approximately 72.4% [4]. The diagnostic criteria for sarcopenia include the use of bioelectrical impedance analysis (BIA), dual-energy X-ray absorptiometry (DXA), or other techniques to quantify muscle mass, along with grip strength or walking speed tests to assess muscle function [5, 6]. Different regions have established specific diagnostic criteria based on the physique and lifestyle habits of the local population, such as those set by the European Working Group on Sarcopenia in Older People (EWGSOP) and the Asian Working Group on Sarcopenia (AWGS) [7, 8]. Sarcopenia leads to various adverse outcomes including falls, fractures, cognitive issues, and reduced quality of life [9–11]. Moreover, sarcopenia is associated with the development and progression of several chronic diseases such as cardiovascular diseases, diabetes, and chronic respiratory diseases [12–14], significantly increasing the demand for social care and support [15]. In the United States, hospitalization costs related to sarcopenia totaled approximately $40.4 billion, with an average cost of $2,600 per patient, significantly higher than for patients without sarcopenia [16]. Therefore, understanding the causes of sarcopenia in older adults is of crucial importance.

The specific pathogenesis of sarcopenia remains unclear, and current discussions predominantly focus on clinical and physiological aspects. Aging is considered the most significant risk factor for sarcopenia, and sensory impairments, which are common age-related conditions, are thought to be related to its pathogenesis [17, 18]. Sensory impairments refer to the reduced or lost ability to perceive stimuli through sensory organs, including vision, hearing, touch, taste, and smell. The most common sensory impairments in the elderly are visual impairment (VI) and HI [19]. Emerging evidence suggests several mechanisms through which sensory impairments may contribute to the development of sarcopenia. Firstly, sensory impairments can lead to decreased physical activity due to mobility challenges and fear of falls, resulting in muscle disuse and atrophy [20, 21]. Secondly, VI and HI may cause social isolation and depression, which are associated with poor nutritional intake and decreased anabolic stimuli for muscle maintenance [22]. Additionally, sensory impairments and sarcopenia may share common pathophysiological pathways, such as chronic inflammation and neurodegeneration, which can simultaneously affect sensory organs and muscle tissue [23, 24].

The link between sarcopenia and sensory impairments remains controversial in the existing literature. For instance, a study in Australia found that women with sensory impairments had an average grip strength 1.1 kg lower [25], while other studies have not found a clear association between VI and weak grip strength [26, 27]. Although these studies have not reached a consensus, they provide a foundation for future research. To date, Cumulative only a few studies have explicitly explored the relationship between specific sensory impairments and sarcopenia [17, 18], with most focusing on subcomponents of sarcopenia in cross-sectional studies [25]. There is currently a lack of sufficient evidence to demonstrate a reliable and significant independent association between the two. Therefore, conducting large-scale prospective cohort studies to thoroughly explore the association between sensory impairments and sarcopenia is essential. We hypothesize that older adults with VI or HI, or both, have a higher risk of developing sarcopenia compared to those without such impairments.

In this study, our goal is to determine whether sensory impairments are a risk factor for sarcopenia. Understanding this could help clinicians improve prevention and intervention measures for sarcopenia. Ultimately, this may help maintain physical function and quality of life in the elderly.

## Materials and methods

### Study population and data source

This longitudinal study utilized data from the CHARLS. CHARLS is a national survey targeting individuals aged 45 and older in China, designed to collect extensive information on the elderly, including socioeconomic status, health conditions, and lifestyle, to thoroughly investigate the lives and health of the elderly population. The CHARLS study has received approval from the Biomedical Ethics Committee of Peking University (IRB00001052-11015). Further details about the CHARLS database can be found in previous publications [28].

Our analysis utilized baseline data from 2011 and follow-up data from 2015. The 2011 baseline survey included 17,708 participants, of whom 13,056 completed the follow-up four years later. We excluded participants younger than 60 years (*n* = 7,418) and those missing key variable data, including education (*n* = 486), marital status (*n* = 282), smoking habits (*n* = 89), drinking habits (*n* = 286), multimorbidity (*n* = 2,165), sensory impairments (*n* = 133), match with CHARLS 2011 data (*n* = 2,076), and sarcopenia (*n* = 578). Ultimately, 4,195participants above the age of 60 were enrolled in this study (Supplementary Fig. 1).

### Sensory impairments

VI refers to partial or complete blindness. We asked respondents to evaluate their ability to see near and distant objects, with options including excellent, very good, good, fair, and poor. Respondents who selected “fair” or “poor” were considered to have a VI; the others were regarded as having no VI. HI refers to partial or complete loss of hearing. Respondents were asked to assess their hearing ability using the same options as for VI. Those who selected “fair” or “poor” were considered to have a HI; the others were regarded as having no HI. If respondents had both VI and HI, they were classified as having DSI.

Based on the sensory impairment status of respondents, we divided the sample into four main categories: (1) NSI, (2) VI only, (3) HI only, and (4) DSI.

It should be noted that the assessment of sensory impairments is based on respondents’ self-reports, which may introduce subjective bias. Therefore, our results should be interpreted in light of its limitations. We further explore the potential impact of this limitation on the study’s conclusions in the discussion section.

### Sarcopenia

The assessment of sarcopenia is based on the revised guidelines of the Asian Working Group on Sarcopenia (AWGS 2019) [7]. This evaluation includes three core components: muscle strength, muscle mass, and physical performance. An individual is considered to have possible sarcopenia if they show low muscle strength or physical performance [7, 29]. The diagnosis of sarcopenia is confirmed when low muscle strength or physical performance coincides with low muscle mass. Therefore, individuals are categorized into three groups based on the severity of sarcopenia (0 = non-sarcopenia; 1 = possible sarcopenia; 2 = confirmed sarcopenia).

Muscle strength measurement: Participants measure grip strength with both dominant and non-dominant hands using a dynamometer, with the highest of two attempts taken for analysis. The criteria for low muscle strength are defined as less than 28 kg for men and less than 18 kg for women.

Appendicular skeletal muscle mass (ASM) assessment: ASM is estimated using a validated bioelectrical equation [30], which is highly consistent with dual-energy X-ray absorptiometry (DXA) [31]. Low muscle mass is defined based on gender and height ratio, typically the lowest 20% of the population’s ASM/Ht^2 value. In this study, low muscle mass is identified as less than 7.07 kg/m^2 for men and less than 5.38 kg/m^2 for women.

Physical performance measurement: This is assessed through gait speed and chair stand tests. Low physical performance is defined as a gait speed of less than 1.0 m/s or a chair stand test time of 12 s or more.

### Covariates

Based on previous studies, we selected sociodemographic characteristics, lifestyle factors, and primary clinical conditions as covariates for our analysis. Sociodemographic characteristics include age, gender (female, male), education level (elementary or below, middle school, high school, college and above), place of residence (rural, urban), and marital status (unmarried, married). Lifestyle factors cover smoking (yes, no) and alcohol consumption (yes, no). Major clinical factors encompass whether the elderly were diagnosed with any of the following conditions: malignancies, psychiatric problems, digestive disease, stroke, heart problems, diabetes, and hypertension. Additionally, we considered fall incidents among the elderly within the past two years (yes, no).

### Statistical analysis

In the descriptive statistical analysis, continuous variables are presented as mean ± standard deviation, while categorical variables are shown in counts and percentages. This study utilizes one-way analysis of variance (ANOVA) and chi-square tests to examine differences in sociodemographic characteristics, lifestyle factors, and clinical features among individuals with various sensory impairments.

In this study, we used an ordered logistic regression model to examine how baseline sensory impairments are related to sarcopenia after four years. This type of regression model is appropriate because sarcopenia is categorized based on severity, which creates an ordered outcome. To avoid the influence of confounding factors, we included different control variables in three separate models. Model 1 included age, gender, education level, place of residence, and marital status. Model 2 added smoking and alcohol consumption. Model 3 further included comorbidities (e.g., cancer, mental health issues, digestive problems, stroke, heart disease, diabetes, hypertension) and fall events. To explore the association between changes in sensory impairments over time and sarcopenia status, we employed a method similar to other studies on sensory impairments [32]. We categorized changes in sensory impairments into nine types: ‘NSI’, ‘NSI→VI’, ‘NSI→HI’, ‘NSI→DSI’, ‘VI→VI’, ‘VI→DSI’, ‘HI→HI’, ‘HI→DSI’, ‘DSI→DSI’, using the ‘NSI’ group as a reference. Based on the same rationale, we analyzed the associations between these changes and sarcopenia using an ordered logistic regression model. Additionally, to understand how sensory impairments are related to specific components of sarcopenia, we analyzed each subcomponent separately. Since these subcomponents are binary (either present or not), we used logistic regression for these analyses. All statistical analyses were conducted using Stata15 software, and a p-value of less than 0.05 was considered statistically significant.

## Results

### Sample characteristics and differences between subgroups

Table 1 displays the baseline characteristics of respondents with different sensory impairment categories and the differences between these groups. Among a total of 4,195 respondents, 1,015 had NSI, 524 had VI, 751 had HI, and 1,905 had DSI, affecting a total of 3,180 individuals with some form of sensory impairment. Statistical analysis revealed significant differences (*p* < 0.05) between the different impairment groups in variables such as age, gender, place of residence, education level, smoking and drinking habits, heart problems, hypertension, fall incidence, muscle strength, muscle mass, and physical performance. After four years of follow-up, the categorization of sensory impairments based on changes over time was further refined into 9 groups.

**Table 1:** Baseline characteristics of respondents and differences between groups

	NSI	VI	HI	DSI	Total	*p*-value
	(*N* = 1015)	(*N* = 524)	(*N* = 751)	(*N* = 1905)	(*N* = 4195)	
Age						< 0.001
Mean (SD)	66.54 (5.94)	67.11 (6.22)	67.62 (6.15)	67.73 (6.13)	67.34 (6.12)	
Gender						< 0.001
Male	540 (53.2%)	229 (43.7%)	412 (54.9%)	888 (46.6%)	2069 (49.3%)	
Female	475 (46.8%)	295 (56.3%)	339 (45.1%)	1017 (53.4%)	2126 (50.7%)	
Census						< 0.001
Rural	785 (77.3%)	421 (80.3%)	581 (77.4%)	1601 (84.0%)	3388 (80.8%)	
Urban	230 (22.7%)	103 (19.7%)	170 (22.6%)	304 (16.0%)	807 (19.2%)	
Education						< 0.001
Elementary school and below	774 (76.3%)	437 (83.4%)	610 (81.2%)	1642 (86.2%)	3463 (82.6%)	
Junior high school	157 (15.5%)	59 (11.3%)	89 (11.9%)	194 (10.2%)	499 (11.9%)	
High school	54 (5.3%)	20 (3.8%)	41 (5.5%)	50 (2.6%)	165 (3.9%)	
College and above	30 (3.0%)	8 (1.5%)	11 (1.5%)	19 (1.0%)	68 (1.6%)	
Marry						0.814
Not married	200 (19.7%)	102 (19.5%)	155 (20.6%)	362 (19.0%)	819 (19.5%)	
Married	815 (80.3%)	422 (80.5%)	596 (79.4%)	1543 (81.0%)	3376 (80.5%)	
Smoking						0.004
No	582 (57.3%)	325 (62.0%)	401 (53.4%)	1146 (60.2%)	2454 (58.5%)	
Yes	433 (42.7%)	199 (38.0%)	350 (46.6%)	759 (39.8%)	1741 (41.5%)	
Drinking						0.015
No	675 (66.5%)	366 (69.8%)	504 (67.1%)	1365 (71.7%)	2910 (69.4%)	
Yes	340 (33.5%)	158 (30.2%)	247 (32.9%)	540 (28.3%)	1285 (30.6%)	
Malignancies						0.229
No	1011 (99.6%)	520 (99.2%)	742 (98.8%)	1885 (99.0%)	4158 (99.1%)	
Yes	4 (0.4%)	4 (0.8%)	9 (1.2%)	20 (1.0%)	37 (0.9%)	
Psychiatric problems						0.178
No	1008 (99.3%)	517 (98.7%)	740 (98.5%)	1873 (98.3%)	4138 (98.6%)	
Yes	7 (0.7%)	7 (1.3%)	11 (1.5%)	32 (1.7%)	57 (1.4%)	
Digestive disease						< 0.001
No	864 (85.1%)	415 (79.2%)	585 (77.9%)	1386 (72.8%)	3250 (77.5%)	
Yes	151 (14.9%)	109 (20.8%)	166 (22.1%)	519 (27.2%)	945 (22.5%)	
Stroke						0.053
No	997 (98.2%)	507 (96.8%)	722 (96.1%)	1843 (96.7%)	4069 (97.0%)	
Yes	18 (1.8%)	17 (3.2%)	29 (3.9%)	62 (3.3%)	126 (3.0%)	
Heart problems						< 0.001
No	891 (87.8%)	473 (90.3%)	628 (83.6%)	1556 (81.7%)	3548 (84.6%)	
Yes	124 (12.2%)	51 (9.7%)	123 (16.4%)	349 (18.3%)	647 (15.4%)	
Diabetes						0.220
No	957 (94.3%)	489 (93.3%)	706 (94.0%)	1761 (92.4%)	3913 (93.3%)	
Yes	58 (5.7%)	35 (6.7%)	45 (6.0%)	144 (7.6%)	282 (6.7%)	
Hypertension						0.001
No	741 (73.0%)	371 (70.8%)	505 (67.2%)	1259 (66.1%)	2876 (68.6%)	
Yes	274 (27.0%)	153 (29.2%)	246 (32.8%)	646 (33.9%)	1319 (31.4%)	
Fall occurrence						< 0.001
No	876 (86.3%)	435 (83.0%)	612 (81.5%)	1448 (76.0%)	3371 (80.4%)	
Yes	139 (13.7%)	89 (17.0%)	139 (18.5%)	457 (24.0%)	824 (19.6%)	
Muscle strength						0.002
No	461 (46.3%)	221 (43.8%)	302 (41.9%)	700 (39.0%)	1684 (41.9%)	
Yes	535 (53.7%)	283 (56.2%)	419 (58.1%)	1096 (61.0%)	2333 (58.1%)	
Physical performance						< 0.001
No	598 (59.9%)	277 (54.1%)	419 (56.8%)	898 (48.5%)	2192 (53.5%)	
Yes	400 (40.1%)	235 (45.9%)	319 (43.2%)	954 (51.5%)	1908 (46.5%)	
Muscle mass						0.014
No	754 (74.3%)	378 (72.1%)	531 (70.7%)	1308 (68.7%)	2971 (70.8%)	
Yes	261 (25.7%)	146 (27.9%)	220 (29.3%)	597 (31.3%)	1224 (29.2%)	

**Fig. 1:**
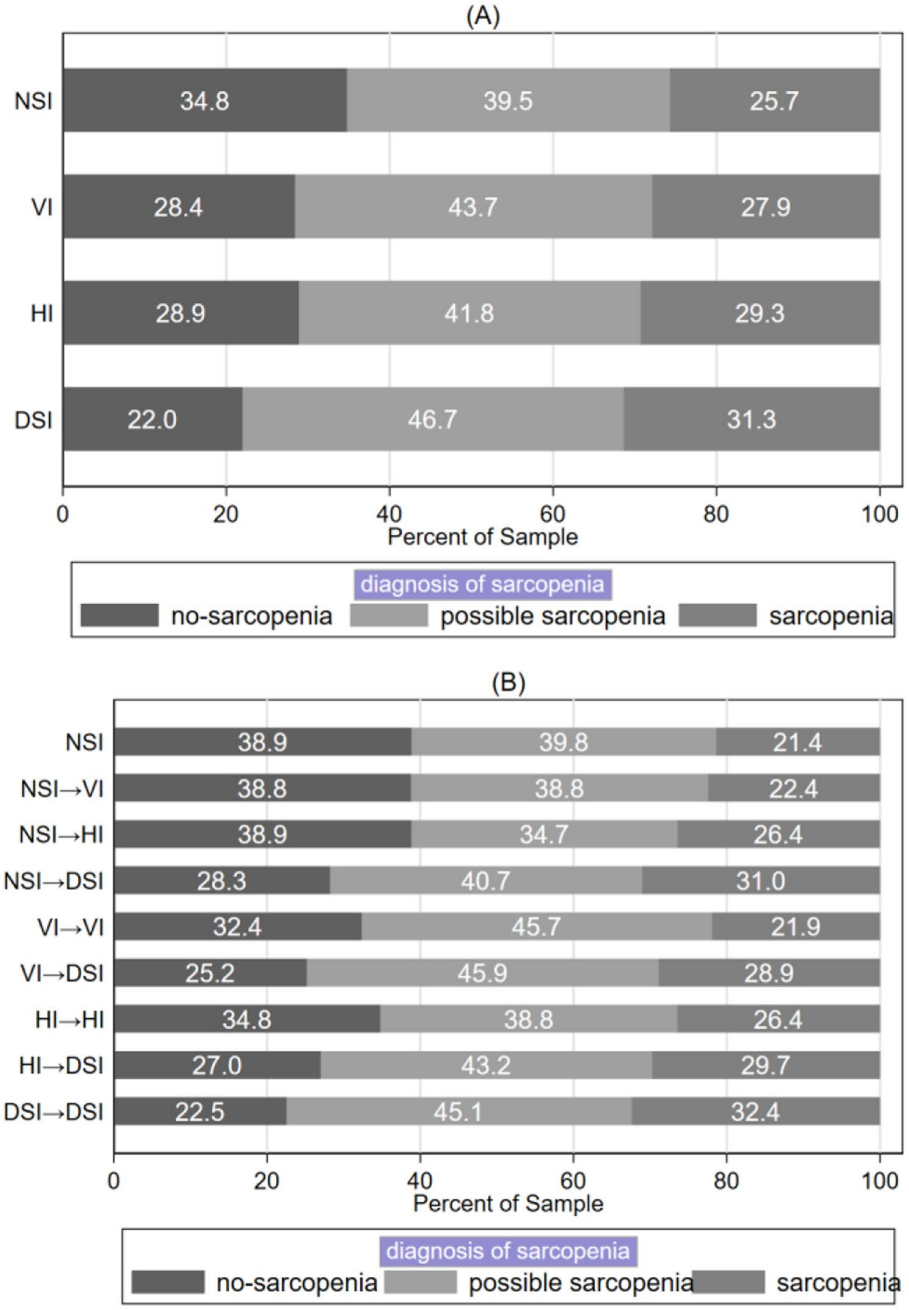
(**A**) Incidence of sarcopenia among respondents with different categories of sensory impairments, (**B**) Incidence of sarcopenia diagnoses among respondents with changes in sensory impairment status

Figure 1A shows the incidence of sarcopenia among respondents with different categories of sensory impairments over a four-year follow-up period. Among those with NSI, 25.7% were diagnosed with sarcopenia; the rate was 27.9% among those with VI, 29.3% among those with HI, and 31.3% among those with DSI. These differences were statistically significant in the chi-square test (*p* < 0.05). Figure 1B shows the distribution of sarcopenia diagnoses among respondents with different types of sensory impairment status changes. The incidence of sarcopenia diagnoses was 21.4%, 22.4%, 26.4%, 31.0%, 21.9%, 28.9%, 26.4%, 29.7%, and 32.4%, respectively. These differences were also statistically significant in the chi-square test (*p* < 0.05).

### The longitudinal association between sensory impairments and Sarcopenia

Table 2 presents the results of ordered logistic regression models examining the association between baseline sensory impairment status and sarcopenia four years later. After adjusting for all covariates (Models 1, 2, and 3), individuals with DSI had a 1.308 times higher risk (95% CI: 1.126–1.519) of developing sarcopenia after four years compared to those with NSI. This indicates that DSI was significantly associated with an increased risk of sarcopenia. In contrast, single sensory impairments (VI and HI) were not significantly associated with sarcopenia. Specifically, compared to individuals with NSI, those with VI or HI had a 1.114 times (95% CI: 0.909–1.366) and 1.107 times (95% CI: 0.922–1.330) higher risk of sarcopenia, respectively. This suggests that VI or HI did not significantly affect sarcopenia in older adults.

**Table 2:** Relationship between baseline sensory impairments and four-year Follow-Up of Sarcopenia

	Model 1		Model 2		Model 3	
	odds ratio	95% CI	odds ratio	95% CI	odds ratio	95% CI
NSI	1 (Ref.)		1 (Ref.)		1 (Ref.)	
VI	1.164	(0.952–1.422)	1.135	(0.927–1.389)	1.114	(0.909–1.366)
HI	1.143	(0.954–1.369)	1.126	(0.939–1.351)	1.107	(0.922–1.330)
DSI	1.416***	(1.224–1.637)	1.335***	(1.152–1.546)	1.308***	(1.126–1.519)
Gender	1.302***	(1.161–1.461)	1.149*	(1.019–1.296)	1.381***	(1.176–1.622)
Age	1.113***	(1.102–1.124)	1.112***	(1.100–1.123)	1.115***	(1.103–1.127)
Census			0.512***	(0.435–0.602)	0.538***	(0.456–0.635)
Education			0.781***	(0.698–0.873)	0.793***	(0.708–0.888)
Marry			0.816**	(0.700–0.951)	0.826*	(0.708–0.964)
Smoking					1.406***	(1.204–1.641)
Drinking					0.882	(0.766–1.016)
Malignancies					1.394	(0.746–2.607)
Psychiatric problems					1.325	(0.806–2.180)
Digestive diseases					1.285***	(1.115–1.481)
Stroke					1.303	(0.939–1.809)
Heart problems					1.112	(0.942–1.312)
Diabetes					1.013	(0.804–1.275)
Hypertension					0.603***	(0.530–0.687)
Fall occurrence					1.071	(0.924–1.242)
Observations	4,195		4,195		4,195	

Furthermore, we conducted a sex-stratified subgroup analysis using Model 3 (Supplementary Table 1). The association between DSI and sarcopenia was statistically significant among female participants but not among male participants. Specifically, in the male group, individuals with DSI had a 1.210 times higher risk (95% CI: 0.980–1.494) of developing sarcopenia after four years compared to those with NSI. In the female group, individuals with DSI had a 1.406 times higher risk (95% CI: 1.135–1.743) of sarcopenia after four years compared to those with NSI. This suggests that sex may influence the association between sensory impairment status and sarcopenia. Therefore, sex-specific interventions might be considered in the prevention and treatment strategies for sarcopenia.

Table 3 utilizes an ordered logistic regression model to demonstrate the association between changes in sensory impairment status over time and sarcopenia. After adjusting for all covariates (Models 1, 2, and 3), individuals who transitioned from NSI to DSI, from HI to DSI, and those with persistent DSI had 1.372 times (95% CI: 1.028–1.830), 1.334 times (95% CI: 1.002–1.778), and 1.470 times (95% CI: 1.159–1.864) higher risks of developing sarcopenia after four years compared to those with persistent NSI. In contrast, other changes in sensory impairment status did not result in significant changes in sarcopenia risk. Specifically, compared to individuals with persistent NSI over four years, those who transitioned from NSI to VI, from NSI to HI, from VI to VI, from VI to DSI, and from HI to HI had risks of sarcopenia that were 0.926 times (95% CI: 0.614–1.396), 0.947 times (95% CI: 0.669–1.340), 0.991 times (95% CI: 0.648–1.513), 1.327 times (95% CI: 0.966–1.824), and 0.972 times (95% CI: 0.699–1.350), respectively. Notably, although the transition from VI to DSI did not reach statistical significance, the trend was close to significant. These results suggest that worsening sensory impairment over time is significantly associated with an increased risk of sarcopenia. Particularly, individuals who transitioned to DSI or had persistent DSI experienced a significant increase in risk; however, those who transitioned to single sensory impairment or had persistent single sensory impairment did not exhibit a significant increase in risk.

**Table 3:** Association between changes in sensory impairment status and Sarcopenia

	Model 1		Model 2		Model 3	
	odds ratio	95% CI	odds ratio	95% CI	odds ratio	95% CI
NSI→NSI	1 (Ref.)		1 (Ref.)		1 (Ref.)	
NSI→VI	1.002	(0.669–1.501)	0.923	(0.612–1.391)	0.926	(0.614–1.396)
NSI→HI	1.049	(0.745–1.477)	0.927	(0.656–1.309)	0.947	(0.669–1.340)
NSI→DSI	1.572**	(1.184–2.086)	1.373*	(1.030–1.829)	1.372*	(1.028–1.830)
VI→VI	0.997	(0.657–1.511)	0.996	(0.654–1.518)	0.991	(0.648–1.513)
VI→DSI	1.548**	(1.134–2.115)	1.341	(0.978–1.838)	1.327	(0.966–1.824)
HI→HI	1.128	(0.817–1.557)	1.016	(0.733–1.407)	0.972	(0.699–1.350)
HI→DSI	1.483**	(1.120–1.964)	1.346*	(1.013–1.789)	1.334*	(1.002–1.778)
DSI→DSI	1.748***	(1.389–2.199)	1.522***	(1.205–1.923)	1.470**	(1.159–1.864)
Gender	1.300***	(1.138–1.484)	1.132	(0.986–1.301)	1.379***	(1.147–1.658)
Age	1.126***	(1.113–1.140)	1.127***	(1.113–1.141)	1.132***	(1.117–1.146)
Census			0.494***	(0.410–0.595)	0.517***	(0.428–0.625)
Education			0.767***	(0.676–0.870)	0.779***	(0.686–0.884)
Marry			0.750**	(0.628–0.896)	0.753**	(0.630–0.901)
Smoking					1.390***	(1.164–1.660)
Drinking					0.970	(0.824–1.142)
Malignancies					1.300	(0.642–2.635)
Psychiatric problems					1.261	(0.733–2.170)
Digestive diseases					1.379***	(1.169–1.625)
Stroke					1.132	(0.778–1.646)
Heart problems					1.163	(0.960–1.409)
Diabetes					1.012	(0.776–1.320)
Hypertension					0.636***	(0.548–0.739)
Fall occurrence					1.093	(0.920–1.298)
Observations	3,193		3,193		3,193	

### Association between sensory impairments and subcomponents of Sarcopenia

**Fig. 2:**
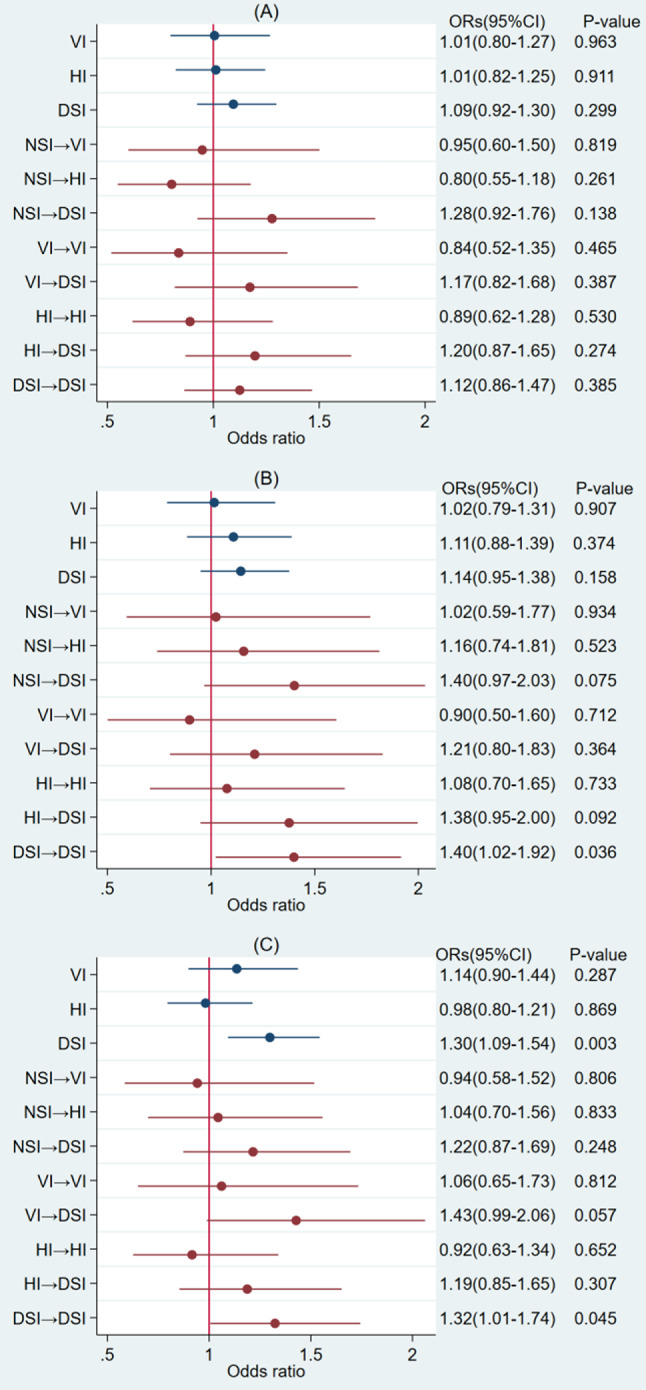
(**A**) The dependent variable is muscle strength, (**B**) The dependent variable is muscle mass, (**C**) The dependent variable is physical performance

Regarding the relationship between sensory impairment and the subcomponents of sarcopenia, our analysis using the full Model 3 indicated that individuals with persistent DSI had a 1.40 times higher risk (95% CI: 1.02–1.92) of low muscle mass compared to those with NSI (Fig. 2B). Additionally, compared to individuals with NSI, those with baseline DSI and persistent DSI had 1.30 times (95% CI: 1.09–1.54) and 1.32 times (95% CI: 1.01–1.74) higher risks of poor physical performance, respectively (Fig. 2C). However, our analysis did not reveal any association between sensory impairment (including VI, HI, DSI, and changes in sensory impairment status) and low muscle strength (Fig. 2A).

## Discussion

In our longitudinal study, we found no correlation between single sensory impairments and sarcopenia (defined as low muscle strength + low physical performance + low muscle mass), a novel finding not previously reported in the literature. However, the literature on sarcopenia defined by different methods does not show consistent results. Some studies have reported an association between single sensory impairments and the development of sarcopenia. For instance, Smith et al. found that individuals with moderate to severe VI had a higher probability of developing sarcopenia compared to those without VI [17]. Harita observed a higher incidence of sarcopenia among individuals with HI in a cross-sectional study of Japanese adults aged 65 and over [18]. Vancampfort noted that individuals with HI were more likely to exhibit weak grip strength [26]. Verghese found that in a four-year study, American adults aged 65 and over with self-reported VI had a significantly increased risk of slow gait [33]. However, other studies have found no direct link between single sensory impairments and sarcopenia. For example, Gopinath’s study observed no significant differences in grip strength between individuals with single sensory impairments and those without [25]. Additionally, two studies found no association between self-reported or objectively measured VI and weak grip strength [26, 27]. In our analysis of the association between sensory impairments and sarcopenia subgroups, we also found similar conclusions; there was no correlation between sensory impairments (including VI, HI, DSI, and changes in sensory impairment status) and low muscle strength.

We believe that these differing conclusions may stem from methodological differences. First, the definitions of sarcopenia vary among studies. Some studies mainly focus on decreased grip strength [17, 25, 26], others emphasize slowed gait [33], and some exclude walking speed from their assessments [18]. Even among studies focusing on decreased grip strength, measurement standards differ. Some define weak grip strength as less than 26 kg for men and less than 18 kg for women [27]. while others use less than 30 kg for men and less than 20 kg for women [26]. Our definition of weak grip strength aligns with that of Vancampfort et al. Definitions of slow gait also vary; some studies define it as walking speed 1 SD below age and sex means [33], while we use a standard of walking speed less than 1.0 m/s. Secondly, the selection of samples shows differences. Different studies included participants from various age groups: two studies targeted individuals aged 50 and above [17, 26], four studies focused on those aged 65 and above [18, 25, 27, 33], and our study included participants aged 60 and above. Additionally, differences in statistical analysis methods may lead to varying results. Some studies treated grip strength as a continuous variable using linear regression analysis [17], while others dichotomized grip strength based on thresholds and used multivariable logistic regression models [27]. In our study, we employed an logistic regression model.

DSI is associated with poor physical performance and muscle mass but not muscle strength. We speculate that this is because muscle strength is primarily influenced by neuromuscular and muscle fiber components, which may not be directly affected by sensory input. Although DSI hinder physical activity and performance, they may not significantly impact the intrinsic contractile properties of muscle tissue. Additionally, the time factor needs to be considered. The decline in muscle strength is a gradual process, and the effect of DSI on muscle strength may only become apparent over a longer time frame. This necessitates future studies with extended follow-up periods to confirm.

Our study also found a significant association between DSI and sarcopenia. Although current research has not directly proven a causal relationship between DSI and sarcopenia, previous studies have suggested from various perspectives that there may be a specific link between sensory impairments and sarcopenia in the elderly population. Notably, compared to single sensory impairments, DSI has a more pronounced impact on the elderly. Similar observations have been made in research on aging-related diseases. Studies indicate that the cumulative number of sensory impairments is proportionally related to the risk of developing dementia, with multiple sensory impairments significantly increasing the risk of dementia [34]. The effects of DSI are considerably more severe than single sensory impairments in terms of daily living activities, social abilities, and mobility, leading to a significant decline in patient life satisfaction [35]. Compared to elderly individuals with single sensory impairments, those with DSI experience more severe cognitive declines [36] and greater negative impacts on health-related quality of life (HRQoL) [37]. In summary, DSI has a more severe adverse effect on the physical functioning of elderly individuals [38].

Single sensory impairment is not associated with sarcopenia, but DSI is associated with it. To explain the differing impacts of single sensory impairment and DSI on sarcopenia, we can refer to the theory of sensory compensation. According to this theory, when one sensory function is impaired, other senses may adapt to compensate for the loss [39]. For example, older adults with VI often rely more on their hearing or touch to navigate their environment, which helps maintain daily functioning and activity levels [40], potentially slowing down the decline of muscle function. A study involving elderly individuals with age-related hearing loss found that those with hearing impairment exhibited a stronger association between visual acuity and cognitive ability, suggesting that visual acuity may play a compensatory role in maintaining cognitive function in the presence of hearing impairment [41]. Although compensatory mechanisms in the elderly may not be as pronounced as in individuals with congenital sensory impairments, they still exist. However, when DSI occurs, these compensatory mechanisms are weakened or lost, leading to greater challenges in maintaining mobility and muscle mass. We also observed a sex-specific association between DSI and sarcopenia. There are significant differences in sarcopenia between males and females, and the predictors of sarcopenia also differ between the sexes [42]. One study suggests that the decline in muscle mass among Asian men may be due to the loss of skeletal muscle, but in women, the decrease in muscle mass is also influenced by body fat and central obesity [43]. These differences between men and women may support the different associations observed between DSI and sarcopenia; however, the specific mechanisms require further exploration.

Furthermore, this study further explored the association between changes in sensory impairments over time and the progression of sarcopenia. We found that transitions from NSI to DSI, from HI to DSI, and persistent DSI were all significantly associated with sarcopenia. These findings emphasize the importance of changes in sensory impairments over time in predicting the risk of sarcopenia and suggest that sensory impairments may have a cumulative negative impact on sarcopenia. Previous research also indicates the cumulative effect of sensory impairments over time, such as how the progression of early sensory impairments could be an early warning sign of neurodegenerative diseases. Studies have shown that monitoring the progression of sensory impairments over time using longitudinal cognitive function assessments can predict the risk of mild cognitive impairment in the elderly [44]. Especially when combined with longitudinal MRI data and machine learning techniques, such dynamic monitoring of sensory impairments can more effectively predict the development of Alzheimer’s disease [45]. The progression of DSI has also been found to be an important predictor of mortality within one year among the elderly [46].

The correlation between sensory impairments and sarcopenia can be explained by several possible mechanisms. First, sensory impairments may affect an individual’s mobility and daily functioning, thereby indirectly leading to a reduction in muscle mass [20, 21]. Second, sensory impairments might impact dietary habits and nutritional intake [22]. For example, a decrease in olfactory or gustatory function can reduce appetite, leading to malnutrition and affecting muscle synthesis and maintenance. Additionally, the degeneration of neurological pathways may play a critical role between sensory impairments and sarcopenia. The loss of vision or hearing reduces the brain’s perception of external stimuli, leading to a lack of sensory input and decreased neuronal activity, which causes the degeneration of these pathways [23]. This degeneration further results in insufficient neural innervation of muscles and a reduction in the central activation signals to muscles, ultimately leading to a decrease in muscle mass and strength [47]. Lastly, some studies suggest that chronic inflammation might simultaneously affect the nervous and muscular systems, potentially linking sensory impairments with sarcopenia [24]. For instance, chronic inflammation could damage sensory nerves and impact the synthesis and repair capabilities of muscles, thereby establishing a potential connection between these two conditions. Overall, the correlation between sensory impairments and sarcopenia may involve multiple physiological and pathological mechanisms, necessitating further research to explore the specific connections and interactions between them in detail.

Our research reveals a close link between sensory impairments and sarcopenia in the elderly, emphasizing the importance of early detection and intervention in preventing the progression of sarcopenia. Therefore, we recommend regular screening for sensory impairments in the elderly on a clinical basis, incorporating assessments of sensory damage into the clinical evaluation of sarcopenia risk as an effective measure to prevent related physical decline. For elderly individuals with DSI, corrective measures such as hearing aids or cataract surgery to address vision and hearing issues may help maintain their mobility and muscle mass. For those with only a single sensory impairment, proactive preventative measures should be taken to prevent the progression to DSI. We advise governments and health departments to invest more resources in promoting high-quality vision and hearing healthcare services for the elderly, including regular auditory and visual checks, economically viable corrective measures, and professional rehabilitation support.

Our study utilizes nationally representative data for longitudinal analysis, which is one of its significant strengths. However, the study also has several limitations. First, self-reported sensory impairments are valuable for capturing individuals’ perceptions of their functional vision and hearing [48]. However, it is important to acknowledge that there may be some discrepancies between self-reported and clinically measured visual and auditory conditions. Studies show that older adults tend to overestimate their VI and underestimate their HI [49, 50]. This reliance on self-reporting may introduce both overestimation and underestimation biases, which could affect the strength of the reported associations. Similar biases have also been observed in other studies using the CHARLS database related to sensory impairments [51]. Therefore, future research could employ objective measurements to examine these associations. Secondly, the estimation of muscle mass is based on a validated formula, but this method has not been validated in populations outside of Asians [30], which may limit the generalizability of the study’s conclusions. Further research could consider adapting the formula for use in other demographic groups or replicating the method in non-Asian populations to confirm its applicability across diverse groups. Lastly, due to data limitations, this study did not control for medication variables in the elderly, but medication use is a potential adverse influencer of sarcopenia [52, 53]. Subsequent studies might consider including this variable for a more accurate assessment.

## Electronic supplementary material

Below is the link to the electronic supplementary material.

Supplementary Material 1

## Data Availability

The datasets used and/or analysed during the current study are available from the corresponding author on reasonable request.

## Abbreviations

CHARLS: China Health and Retirement Longitudinal Study
AWGS: Asian Working Group on Sarcopenia
ORs: odds ratios
CI: confidence intervals
NSI: No Sensory Impairments
VI: Visual Impairments
HI: Hearing Impairments
DSI: Dual Sensory Impairments
