# Supplementary material for: Association between sensory impairment and sarcopenia in older Chinese adults: a 4-Year longitudinal study
Source: BMC Geriatr. 2025 Feb 11;25:90. doi: 10.1186/s12877-024-05642-6 (PMC11817050; doi:10.1186/s12877-024-05642-6)
Supplement: Supplementary file 1 — Supplementary Material 1 [file 12877_2024_5642_MOESM1_ESM.docx]

# supplementary material

**Supplementary Figure 1. The Sample Selection in CHARLS**

**Supplementary Table 1: Subgroup Analysis by Gender**

|  | Model 3: Male group | | Model 3: Female group | |
| --- | --- | --- | --- | --- |
|  | odds ratio | 95% CI | odds ratio | 95% CI |
| NSI | 1 (Ref.) |  | 1 (Ref.) |  |
| VI | 1.117 | (0.832 - 1.499) | 1.116 | (0.840 - 1.482) |
| HI | 1.223 | (0.953 - 1.570) | 0.969 | (0.739 - 1.271) |
| DSI | 1.210 | (0.980 - 1.494) | 1.406** | (1.135 - 1.743) |
| Age | 1.131*** | (1.113 - 1.148) | 1.101*** | (1.086 - 1.118) |
| Census | 0.519*** | (0.415 - 0.651) | 0.561*** | (0.438 - 0.718) |
| Education | 0.846* | (0.740 - 0.967) | 0.677*** | (0.545 - 0.843) |
| Marry | 0.820 | (0.632 - 1.064) | 0.796* | (0.655 - 0.968) |
| Smoking | 1.365** | (1.129 - 1.650) | 1.591*** | (1.213 - 2.086) |
| Drinking | 0.895 | (0.756 - 1.061) | 0.904 | (0.697 - 1.173) |
| Malignancies | 1.370 | (0.429 - 4.373) | 1.493 | (0.703 - 3.170) |
| Psychiatric problems | 1.615 | (0.725 - 3.597) | 1.112 | (0.587 - 2.107) |
| Digestive diseases | 1.334** | (1.083 - 1.642) | 1.248* | (1.026 - 1.519) |
| Stroke | 1.240 | (0.795 - 1.935) | 1.336 | (0.818 - 2.182) |
| Heart problems | 1.097 | (0.860 - 1.399) | 1.102 | (0.876 - 1.385) |
| Diabetes | 1.270 | (0.897 - 1.797) | 0.860 | (0.631 - 1.173) |
| Hypertension | 0.589*** | (0.487 - 0.712) | 0.618*** | (0.516 - 0.740) |
| Fall occurrence | 1.010 | (0.807 - 1.264) | 1.114 | (0.914 - 1.358) |
| Observations | 2,069 |  | 2,126 |  |
